# Copy number expansion of the *STX17* duplication in melanoma tissue from Grey horses

**DOI:** 10.1186/1471-2164-13-365

**Published:** 2012-08-02

**Authors:** Elisabeth Sundström, Freyja Imsland, Sofia Mikko, Claire Wade, Snaevar Sigurdsson, Gerli Rosengren Pielberg, Anna Golovko, Ino Curik, Monika H Seltenhammer, Johann Sölkner, Kerstin Lindblad-Toh, Leif Andersson

**Affiliations:** 1 Department of Animal Breeding and Genetics, Swedish University of Agricultural Sciences, Box 597, SE-751 24, Uppsala, Sweden; 2 Science for Life Laboratory Uppsala, Department of Medical Biochemistry and Microbiology, Uppsala University, Box 582, SE-751 23, Uppsala, Sweden; 3 Department of Animal Breeding and Genetics, Swedish University of Agricultural Sciences, Box 7023, SE-750 07, Uppsala, Sweden; 4 Broad Institute of Harvard and MIT, 7 Cambridge Center, Cambridge, MA, 02142, USA; 5 Center for Human Genetic Research, Massachusetts General Hospital, Boston, MA, 02114, USA; 6 Faculty of Veterinary Sciences, University of Sydney, Sydney, New South Wales, 2006, Australia; 7 Animal Science Department, Faculty of Agriculture, University of Zagreb, Zagreb, HR-10000, Croatia; 8 Clinical Department of Small Animal Surgery, University of Veterinary Medicine, Vienna, Austria; 9 Department of Sustainable Agricultural Systems, University of Natural Resources and Applied Life Sciences, Vienna, A-1180, Vienna, Austria

**Keywords:** *STX17*, Melanoma, Hair greying, Copy number variation, Melanocytes

## Abstract

**Background:**

Greying with age in horses is an autosomal dominant trait, associated with loss of hair pigmentation, melanoma and vitiligo-like depigmentation. We recently identified a 4.6 kb duplication in *STX17* to be associated with the phenotype. The aims of this study were to investigate if the duplication in Grey horses shows copy number variation and to exclude that any other polymorphism is uniquely associated with the *Grey* mutation.

**Results:**

We found little evidence for copy number expansion of the duplicated sequence in blood DNA from Grey horses. In contrast, clear evidence for copy number expansions was indicated in five out of eight tested melanoma tissues or melanoma cell lines. A tendency of a higher copy number in aggressive tumours was also found. Massively parallel resequencing of the ~350 kb Grey haplotype did not reveal any additional mutations perfectly associated with the phenotype, confirming the duplication as the true causative mutation. We identified three SNP alleles that were present in a subset of Grey haplotypes within the 350 kb region that shows complete linkage disequilibrium with the causative mutation. Thus, these three nucleotide substitutions must have occurred subsequent to the duplication, consistent with our interpretation that the Grey mutation arose more than 2,000 years before present.

**Conclusions:**

These results suggest that the mutation acts as a melanoma-driving regulatory element. The elucidation of the mechanistic features of the duplication will be of considerable interest for the characterization of these horse melanomas as well as for the field of human melanoma research.

## Background

Greying with age in horses is an autosomal dominant trait, associated with a gradual loss of hair pigmentation, a high incidence of melanoma and vitiligo-like depigmentation. The phenotype can be traced back through thousands of years in European and Asian culture and throughout history the white horse has been commonly used to manifest purity, power and status. Consequently, humans have favoured the phenotype in breeding over hundreds of generations. Still today, white-coated horses are preferred in many breeds all over the world, particularly notably in the Lipizzaner breed used in the world famous Spanish Riding School in Vienna.

**Figure 1:**
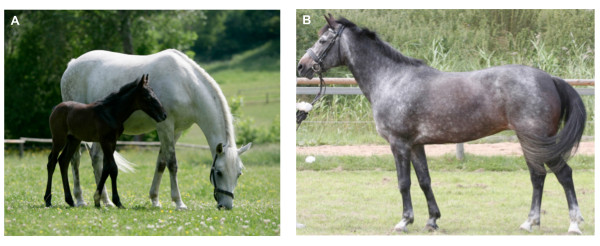
**The Grey horse phenotype.** (**A**) Grey horse with a dark foal. Photo: Meike Pachner. (**B**) A late greying Connemara horse, showing only very few signs of hair greying by the age of 14 years. Photo: Jenny Hagenblad.

Grey horses are born coloured and gradually lose their hair pigmentation (Figure 1A). Most individuals in breeds where the *Grey* allele is common, like Arabian, Andalusian and Lipizzaner horses, become white by the age of 6–8 years [1,2], and sometimes even earlier. The pigmentation loss only affects the hair, while the skin stays dark throughout life, indicating different cellular fates for the melanocytes in the hair follicles and in the skin. Melanomas are frequently occurring among many Grey horses, usually in the later stages of life. It is generally claimed that 70-80% of Grey horses older than 15 years have melanomas [3,4]. The primary tumours arise in the dermis of the glabrous skin, often around the eyes, genital regions or the anus, and are usually benign. Internal tumours do occur but are fairly rare, and it has not been confirmed whether these tumours are true metastases or locally occurring from melanocytes residing in respective tissues. Grey horses with melanomas also develop vitiligo-like skin depigmentation, commonly seen on the muzzle and under the tail [1], and many Grey horses have pigmented speckles in their white fur, a phenotype known as flea-bitten Grey.

In 2008, we identified the mutation causing Greying with age in horses, constituting a 4.6 kb intronic duplication in the *STX17* gene [2]. The mutation resides on a 352 kb haplotype showing complete linkage disequilibrium (LD) with the Grey phenotype across eight breeds. This interval was unexpectedly large since it was deduced from material including both Arabian and Icelandic horses, two divergent breeds that have been separated for a minimum of 1,000 years. As also indicated in a previous study [5], this suggests a low rate of recombination in the Grey interval. Additionally, one non-Grey haplotype identical to the Grey haplotype for all tested SNPs in the interval was suggested to represent the “ancestral” haplotype, or at least a haplotype closely related to Grey [2].

While the loss of hair pigmentation is fully dominant, the speed of greying, amount of speckling, incidence of melanomas and presence of vitiligo-like depigmentation show considerable variation among Grey horses. Horses homozygous for the mutation show a more rapid greying process and tend to become whiter than heterozygous Grey horses [2]. Homozygous individuals also show a significantly higher incidence of melanoma and vitiligo, while they almost lack the pigmented speckles present in heterozygous individuals [2]. However, there is still considerable phenotypic variation within the *G/g* and *G/G* genotypes both within and across breeds with respect to the manifestation of these traits. For example, some individuals in the Connemara breed carrying the *Grey* mutation show an extraordinarily slow rate of greying (Figure 1B). These horses show relatively few signs of greying as late as by 15 years of age, and many of them never turn completely grey. The slow rate of greying appears to be inherited within families, although the molecular reason remains unknown. Possible causes could be an alternative allele of *Grey* in these horses, or genetic variation elsewhere in the genome affecting the penetrance of *Grey*. Quantitative genetic analysis of the speed of greying in the Lipizzaner population (unpublished) indicates that a large genetic component, almost 60 percent of the phenotypic variation as regards how quickly a Grey horse become white (i.e. the speed of greying), is still unexplained.

A copy number variant (CNV) is usually defined as a region larger than 1 kb where copy number differences have been observed between two or more genomes [6]. Duplicated loci often show variation in copy number, which in turn may have important effects on the phenotype. This can be exemplified by the Pea-comb phenotype in chicken, where a massive and variable amplification of a duplicated sequence in intron 1 of *SOX5* results in a dramatically reduced size of the comb and wattles [7], and the Dominant white phenotype in pigs, where a 450 kb duplication, or in some individuals triplication, in *KIT* causes white coat colour [8-10]. In humans, CNVs that often include large regions spanning several genes, are an important aspect of genetic variation, and have been associated with various diseases [11,12]. The role of CNVs in cancer is an emerging field [13]. Acquired, or somatic, copy number alterations (CNAs) in tumour DNA have for instance been identified in lung adenocarcinoma [14] and pediatric acute lymphoblastic leukaemias [15]. Copy number variants in melanocytic tumours have also been investigated, showing that melanomas and benign melanocytic nevi have different patterns of chromosomal aberrations [16].

In the present study, we have investigated whether the duplication in *STX17* shows copy number variation among Grey horses. The results showed a difference in copy number of the duplication between blood and tumour DNA, with the highest copy number occurring in tumours classified as aggressive or derived from horses that were euthanized due to internal tumours. We also used sequence capture and next-generation sequencing to resequence the entire 352 kb *Grey* haplotype in order to exclude the possibility that other polymorphisms show as complete association to the phenotype as the *STX17* duplication and may potentially contribute to the phenotype. We did not find any other mutation showing such a complete association with the Grey phenotype and thus the duplication in *STX17* can be said to be well established as being causative for Greying with age.

## Results

### Amplification of the duplication frequently occurs in tumour DNA but not in germline

**Figure 2:**
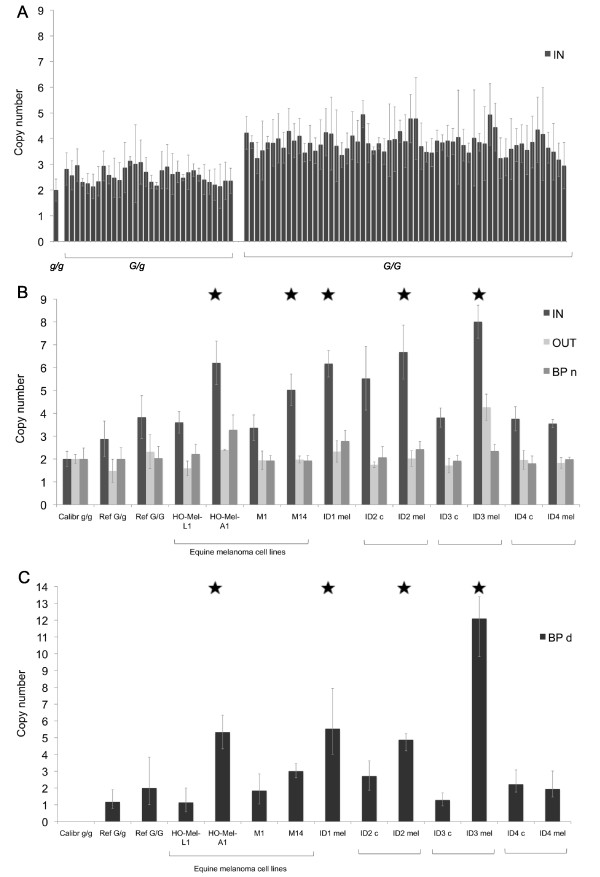
**Copy number variation of the *STX17* duplication.** (**A**) Copy number assay for the *STX17* duplication in constitutional DNA from 94 Grey Lipizzaner horses and one calibrator sample with a known copy number of 2. Mean copy number *G/g* = 2.58, *G/G* = 3.84, SEM (standard error of the mean) *G/g* = 0.05, *G/G* = 0.05. (**B**) Copy number assay for the *STX17* duplication in constitutional DNA and/or melanoma DNA (c = constitutional DNA, mel = melanoma DNA) using three different probes; IN = inside the duplicated sequence, OUT = outside the duplicated sequence and BP n = the border between the 5’flanking sequence and the 5’end of the duplicated sequence. The sample denoted ‘Calibr’ is a *g/g* individual with a known copy number of 2, used as a calibrator in the analysis. Constitutional DNA from one *G/g* and one *G/G* horse was tested in the assay and the results are shown as a reference for the copy number expected from each genotype. Brackets surround the equine melanoma cell lines or the paired constitutional and melanoma DNA samples. Error bars represent the copy number range from the CopyCaller™ Software analysis of quadruplicates in each assay. Samples showing a copy number expansion are marked with an asterisk. (**C**) Copy number assay for the *STX17* duplication breakpoint in constitutional DNA and/or melanoma DNA (c = constitutional DNA, mel = melanoma DNA) using the probe BP d = over the duplication breakpoint. The *G/G* reference sample was used as a calibrator in the analysis. Error bars represent the copy number range from the CopyCaller™ Software analysis of quadruplicates in each assay.

Ninety-four homo- and heterozygous Grey Lipizzaner horses were tested for the copy number of the duplicated sequence in constitutional DNA using a TaqMan copy number assay. The results showed the expected clustering of copy number according to zygosity for *Grey*, and the clear separation of the *Grey* genotype groups did not indicate a frequent variation in copy number (Figure 2A). Analysis of tumour DNA from three horses, of which two were euthanized due to numerous internal melanomas (Table 1), and in two equine melanoma cell lines originating from aggressive tumours (Ho-Mel-A1, M14), revealed a striking expansion of the duplicated sequence, estimated to 5–8 copies (marked by an asterisk in Figure 2B).

**Table 1:** **Samples used in the TaqMan Copy Number Assay for the *Grey* locus**

**ID**	**Grey**	**Type**^**2**^	**Breed**	**Comments**
Calibrator	*g/g*	C_DNA	Arabian	Calibrator in analysis
Ref	*G/g*	C_DNA	Thoroughbred	Genotype reference
Ref	*G/G*	C_DNA	Lipizzaner	Genotype reference
SP1	*G/g*^*1*^	C_DNA	Connemara	Late greying phenotype
SP2	*G/g*^*1*^	C_DNA	Connemara	Late greying phenotype
SP3	*G/g*^*1*^	C_DNA	Connemara	Late greying phenotype
Ho-Mel-L1	*G/g*	Equine melanoma cell line	Lipizzaner	Normal growth
Ho-Mel-A1	*G/g*	Equine melanoma cell line	Arabian	Fast growth
M1	*G/g*	Equine melanoma cell line	Irish warmblood	Normal growth
M14	*G/G*	Equine melanoma cell line	Andalusian	Fast growth
ID1	*G/g*	tumour DNA	Thoroughbred	Euthanized due to melanoma tumours
ID2	*G/G*	C_DNA + tumour DNA	Shagya Arabian	Euthanized due to melanoma tumours
ID3	*G/g*	C_DNA + tumour DNA	Swedish warmblood	Euthanized due to melanoma tumours
ID4	*G/g*	C_DNA + tumour DNA	Connemara	Cremello coloured^3^, euthanized due to laminitis, very small melanomas

^1^ Not genotyped with Grey long-range PCR assay due to fragmented DNA; inferred genotype from copy number assay.

^2^ C_DNA = constitutional genomic DNA from blood or hair samples.

^3^ Cremello horses are homozygous for a missense mutation D153N in SLC45A2 (MATP) and show a severe dilution of pigmentation in hair, skin and eye [25].

Three different probes were initially used in the TaqMan assay (Figure 2B); ** *BP n* ** located at the 5' border of the duplicated sequence, which is expected to be single copy if the expansion only involves the 4.6 kb tandem duplication; ** *IN* ** located within the duplicated sequence; and ** *OUT* ** located 1.5 kb downstream of the duplication, also expected to be a single copy if the expansion only involves the tandem duplication. All samples, except one, showed a normal copy number of 2.0 for both the probe at the 5’border of the duplication (BP n) and the probe outside (OUT) the duplication (Figure 2B and Additional file 1), implying that the higher copy number in tumours represents a specific amplification of the duplicated sequence rather than amplifications of a larger genomic region as often observed in tumour DNA. Only one tumour DNA sample, from individual ID3, showed a slightly higher copy number for one of the flanking probes (Figure 2B). When the region was sequenced by Sanger sequencing, no polymorphism could be detected in the probe site that would explain a lower binding affinity of the TaqMan probe, and hereby a false score in the analysis. To further corroborate the interpretation that the higher copy number in some tumours is caused by a local amplification of the entire tandem duplication we added a fourth TaqMan probe (** *BP d* **; Figure 2C) located exactly at the unique breakpoint between the two tandem copies on Grey chromosomes, this probe is expected to be present in n-1 copies if there is a local amplification of the entire duplicated sequence. The results for four out of the five tumour samples were fully consistent with a local amplification of the duplicated sequence (Figure 2C).

One non-aggressive tumour and constitutional DNA from the same individual (ID4), showed an equal copy number in both tumour and constitutional DNA, close to the expected three copies for a heterozygous Grey horse. DNA from two non-aggressive horse melanoma cell lines (Ho-Mel-L1, M1) also yielded a copy number of 3.0, in accordance with their *Grey* genotype determined by a diagnostic long-range PCR test.

The late greying Connemara horses showed the expected heterozygote copy number of three for the duplicated sequence, one for the duplication breakpoint and two copies of the flanking sequences (Additional file 2), verifying that these horses do indeed carry the Grey duplication.

### The Grey duplication is confirmed by coverage analysis of targeted resequencing

Two individuals were chosen for resequencing of the 352 kb region surrounding the *Grey* locus, one Lipizzaner horse homozygous for *Grey* and one non-Grey chestnut Arabian horse carrying the closely related haplotype, a haplotype identical to *Grey* for all tested polymorphisms except for the duplication [2]. This experimental design was chosen to minimize the number of sequence polymorphisms identified as uniquely associated with *Grey* in this limited sample of resequenced horses but being non-causative. In addition, five other non-Grey horses were included in the experiment.

**Figure 3:**
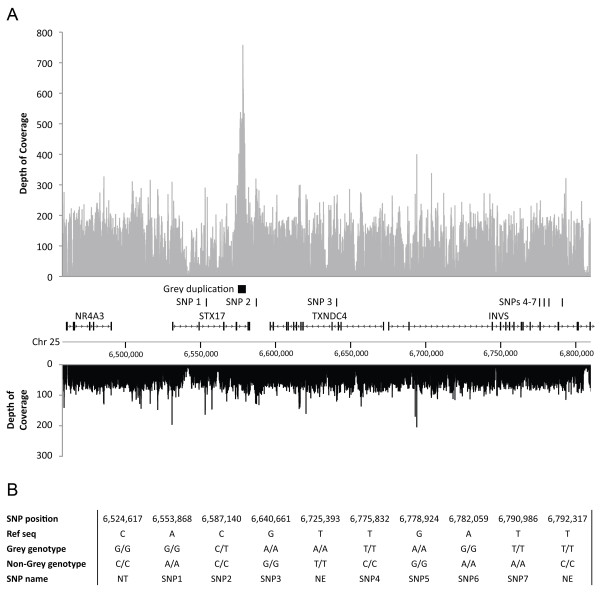
**Targeted resequencing of the Grey haplotype.** (**A**) Depth of coverage given by sequences obtained from targeted resequencing experiment aligned to horse genome reference sequence (EquCab2) confirms the *Grey* duplication in *STX17*; the region presented is the one that is essentially identical-by-descent among all Grey horses tested so far. Upper graph: Grey horse (ID L147, *G/G*). Middle: Chromosomal coordinates, with genes and the *Grey* duplication indicated. SNPs investigated in detail are marked by numbers 1–7. Lower graph: Non-Grey horse (ID 800, *g/g*). Data derived from 75 bp non-sliding windows. (**B**) SNPs detected by targeted resequencing and found to be unique to the homozygous Grey Lipizzaner horse L147, or homozygous for a non-reference allele in all six non-Greys, including individual 800 having a haplotype closely related to the Grey haplotype. The Grey genotype is based on the genotype of the Lipizzaner horse L147. The non-Grey genotype is based on the genotype of all sequenced non-Grey horses. SNPs were selected for further analysis based on Sanger sequencing of Twilight (the Grey heterozygote used to generate the horse reference sequence), one additional Grey homozygote and one horse homozygous non-Grey carrying a haplotype closely related to *Grey*. Abbreviations: NT: Not tested for technical reasons, SEL: Selected for further analysis, SS: Sanger sequencing, NE: Not exclusive to Grey.

The read depth varied considerably between the samples, with good to exceptionally good coverage obtained for the homozygous Grey Lipizzaner, the non-Grey Arabian and three other non-Grey horses, with two non-Grey samples showing adequate coverage for SNP calling, but not as impressive as for the rest (Table 2). This heterogeneity can in all likelihood be attributed to differences in the efficiency of enrichment between the samples (data not shown). The duplication associated with the Grey phenotype was readily observed when read depth was examined for the Grey individual across the whole resequenced region (Figure 3A).

**Table 2:** **Statistics for alignment of sequence reads from the 352 kb region associated with the *Grey* allele**

		**Breed**						
		**Lipizzaner**	**Arabian**	**Noriker**	**Icelandic**	**Knabstrupper**	**Appaloosa**	**Quarter Horse**
** *Grey* genotype**		** *G/G* **	** *g/g* **	** *g/g* **	** *g/g* **	** *g/g* **	** *g/g* **	** *g/g* **
Reads	Av. cover	96	52	7	102	29	10	101
SNPs	Homozygous	101	103	133	422	67	18	100
	Heterozygous	6	13	6	3	247	47	145
	Total	107	116	139	425	314	65	245

Data from eight horses representing seven different breeds were compared with the horse genome reference sequence (Sep. 2007 Broad/EquCab2 assembly). SNP calling was performed based on alignments with the horse reference sequence.

SNP calling was performed on alignments to the repeat-masked reference sequence (Table 2). We identified more than 750 SNPs in the region, in average 2 SNPs/kb, but the homozygous Grey horse carried a unique allele not present in the non-Grey horses at only 15 loci. The Grey homozygous Lipizzaner horse was scored as heterozygous at six of these loci, but Sanger-based resequencing revealed that all these putative heterozygous positions, except one at position 6,587,140 bp, were false positives. For 7 of the 10 remaining loci, the Grey homozygous Lipizzaner horse was either heterozygous or homozygous for the non-reference allele whereas the six non-Grey horses were homozygous for the non-reference allele at the remaining three SNPs (Figure 3B). The thoroughbred mare, named Twilight, from which the genome sequence was derived, is heterozygous at the *Grey* locus [17]. Thus, we postulated that the seven SNPs unique to the Grey Lipizzaner horse are in an area derived from her *non-Grey* allele, whereas the three non-Grey SNPs are in an area where the genome sequence is derived from Twilight's *Grey* allele.

### The duplication is the only polymorphism uniquely associated with the Grey phenotype

The 10 SNPs uniquely associated with the Grey homozygote in the resequencing analysis were further investigated by Sanger sequencing of three additional horses; Twilight, the heterozygous horse used to generate the reference sequence, a second homozygous Grey Lipizzaner horse and a second *g/g* non-Grey animal carrying a haplotype closely related to Grey. Two loci were excluded because the allele was also detected on a non-Grey haplotype and we failed to resequence one of these SNPs (Figure 3B). This left seven loci that were selected for further analysis.

TaqMan SNP assays were successfully designed for five of the remaining SNPs (SNP1, SNP2, SNP3, SNP4 and SNP6; Figure 3B and Table 3) and these were genotyped in 357 Grey and non-Grey horses from 8 different breeds to reveal their association pattern to the Grey phenotype. The genotyping assays for SNP5 and SNP7 failed and those two SNPs were therefore genotyped in key individuals using Sanger sequencing (Table 3).

**Table 3:** TaqMan genotyping of SNPs in the Grey region

			**Polymorphism**^**1**^								
**ID**	**Breed**	**Grey**	**SNP0**	**SNP1**	**Dup**	**SNP2**	**SNP3**	**SNP4**	**SNP5**	**SNP6**	**SNP7**
231	Arabian	*g/g*		A	-	C	G	C		A	
446	Thoroughbred	*g/g*		A	-	C	G	C		A	
1107	New Forest Pony	*g/g*		A	-	C	G	C		A	
1268	Icelandic	*g/g*		A	-	C	G	C		A	
2857	Connemara	*g/g*		A	-	C	G	C		A	
5772	Shetland	*g/g*		A	-	C	G	C		A	
S062	Lipizzaner	*g/g*		A	-	C	G	C		A	
6605	Shetland	*g/g*		A	-	C	G	T/C	A/G	G/A	A
1270	Icelandic	*g/g*		A	-	C	-	T/C	A/G	G/A	A
435	Arabian	*g/g*		A	-	C/T*	G	C		A	
456	Arabian: Anc	*g/g*		A	-	C/T	G	C		A	
800	Arabian: Anc	*g/g*		A	-	C/T	G	C	A	A	A
6972	Welsh	*g/g*		A	-	C/T	G	C		A	
D011	Lipizzaner	*g/g*		A	-	C/T*	G	C		A	
S046	Lipizzaner	*g/g*		A	-	C/T	G	C		A	
6942	New Forest Pony	*G/g*		A	+/−	C	G	C	G	A	A
S057	Lipizzaner	*G/g*		A	+/−	C	G	C	G	A	A
899	Arabian	*G/g*		A	+/−	C	A/G	T/C	A/G	A or G/A	A/T
222	Arabian	*G/g*		A	+/−	C	A/G	T/C		G/A	
5726	Connemara	*G/g*		A	+/−	C	A/G	T/C		G/A	
6591	Connemara	*G/g*		A	+/−	C	A/G	T/C		G/A	
D081	Lipizzaner	*G/g*		A	+/−	C	A/G	T/C		G/A	
D076	Lipizzaner	*G/g*		A/G	+/−	C	A/G	T/C		G/A	
213	Arabian	*G/g*		A	+/−	C/T	A/G	T/C		G/A	
D091	Lipizzaner	*G/g*		A	+/−	C/T	A/G	T/C		G/A	
D007	Lipizzaner	*G/g*		A/G	+/−	C/T	A/G	T/C		G/A	
D010	Lipizzaner	*G/g*		A/G	+/−	C/T*	A/G	T/C		G/A	
678	Arabian	*G/g*		A	+/−	C/T	G	T/C		G/A	
D004	Lipizzaner	*G/g*		A	+/−	C/T	G	T/C		G/A	
452	Thoroughbred	*G/g*		A	+/−	C	G	T/C		G/A	
3156	Lipizzaner	*G/g*		A	+/−	C	G	T/C		G/A	
6577	Welsh	*G/g*		A	+/−	C	G	T/C		G/A	
6611	Shetland	*G/g*		A	+/−	C	G	T/C		G/A	
SP1	Connemara:Sp	*G/g*		A	+/−	C	G	T/C		G/A	
SP2	Connemara:Sp	*G/g*		A	+/−	C	G	T/C		G/A	
SP3	Connemara:Sp	*G/g*		A	+/−	C	G	T/C		G/A	
SP4	Connemara:Sp	*G/g*		-	+/−	C	G	T/C		G/A	
D192	Lipizzaner	*G/g*		A/G	+/−	C	G	T/C		G/A	
1271	Icelandic	*G/g*		A	+/−	C	G	T	A	G	A/T
P023	Lipizzaner	*G/g*	A/T	A/G	+/−	C	A/G	T/C		G/A	
S065	Lipizzaner	*G/g*	A/T	A/G	+/−	C	-	T/C		G/A	
P038	Lipizzaner	*G/g*	A/T	A/G	+/−	C	A/G	T/C		G/A	
L034	Lipizzaner	*G/g*	A/T	A/G	+/−	C	A/G	-		G/A	
P076	Lipizzaner	*G/g*	A/T	A/G	+/−	C	A/G	T/C		G/A	
L040	Lipizzaner	*G/g*	A/T	A/G	+/−	C/T	A/G	T/C		G/A	
L049	Lipizzaner	*G/g*	A/T	A/G	+/−	C/T	A/G	T/C		G/A	
D007	Lipizzaner	*G/g*	A/T	A/G	+/−	C/T	A/G	T/C		G/A	
230	Arabian	*G/G*		A	+/+	C	A	T		G	
L010	Lipizzaner	*G/G*		A	+/+	C	A	T		G	
D080	Lipizzaner	*G/G*		A/G	+/+	C	A	T		G	
D006	Lipizzaner	*G/G*		G	+/+	C	A	T		G	
399	Arabian	*G/G*		A	+/+	C/T	A	T		G	
857	Arabian	*G/G*		A	+/+	C/T*	A	T		G	
L004	Lipizzaner	*G/G*		A	+/+	C/T	A	T		G	
D089	Lipizzaner	*G/G*		A/G	+/+	C/T	A	T		G	
D098	Lipizzaner	*G/G*		G	+/+	C/T	A	T		G	
6210	Lipizzaner	*G/G*		A	+/+	C	A/G	T		G	
D009	Lipizzaner	*G/G*		A/G	+/+	C	A/G	T		G	
D079	Lipizzaner	*G/G*		A	+/+	C/T	A/G	T		G	
P086	Lipizzaner	*G/G*		A	+/+	C/T*	A/G	T		G	
D003	Lipizzaner	*G/G*		A/G	+/+	C/T	A/G	T		G	
L053	Lipizzaner	*G/G*		A/G	+/+	C/T*	A/G	T		G	
L024	Lipizzaner	*G/G*		A	+/+	C	G	T		G	
6580	Welsh	*G/G*		A	+/+	C/T	G	T		G	
D102	Lipizzaner	*G/G*		A	+/+	C/T	G	T		G	
L090	Lipizzaner	*G/G*		A	+/+	C/T*	G	T		G	

Results from TaqMan genotyping of selected SNPs located in the *Grey* region in 357 non-Grey and Grey horses from 8 different breeds, including the late-greying Connemara horses (Connemara:Sp) and Arabian horses (Arabian: Anc) carrying a non-Grey haplotype that is ancestral or closely related to the Grey haplotype. The table shows representative genotypes selected from all breeds. For SNP2, individuals with a strong signal for the variant T allele (C/T ratio equal 1) are marked with an asterisk. SNP0 SNP5 and SNP7 were genotyped by Sanger sequencing only in key individuals. SNP0 is at nucleotide position 6,553,858 in STX17.

^1^SNP1 is at nucleotide position 6,553,868 in *STX17***;** Dup is the 4.6 kb duplication in *STX17* causing Grey; SNP2 is at nucleotide position 6,587,140 in *STX17***;** SNP3 is at nucleotide position 6,640,661 in *TXNDC4*; SNP4 is at nucleotide position 6,775,832 in *INVS*; SNP5 is at nucleotide position 6,778,924 in *INVS*; SNP6 is at nucleotide position 6,782,059 in *INVS*; SNP7 is at nucleotide position 6,790,986 in *INVS***.**

The non-reference alleles at SNP1 and SNP3 were only found in a fraction of Grey individuals (Table 3), which means that these SNPs must have occurred subsequent to the *Grey* mutation. SNP1 is located in a vertebrate conserved element of approximately 20 bp (UCSC Genome Browser; Human Feb. 2009 GRCh37/hg19 Assembly), with a high regulatory potential score (UCSC Genome Browser; Human Mar. 2006 NCBI36/hg18 Assembly), while SNP3 is not. Possibly, due to its location in a conserved element, SNP1 could be functionally important, but this was not further investigated in the present study. For SNP1, a fourth, distinct separate genotype cluster was detected in the TaqMan analysis. By sequencing the amplicon for the individuals in this cluster, a SNP in the TaqMan probe site was detected, resulting in a lower *G*-allele signal in the assay, thereby creating a fourth cluster. Since the *G* nucleotide is associated with *Grey*, this analysis revealed a third confirmed SNP at position 6,553,858 bp, (SNP0 in Table 3) which must have occurred on the *Grey* haplotype subsequent to the Grey mutation event.

SNP4 and SNP6 showed a nearly perfect association with the *Grey* genotype, but with the exception of two non-Grey and four heterozygous Grey individuals (Table 3). For these individuals the SNP4, SNP6 and the *Grey* duplication statuses were confirmed by Sanger sequencing or long-range PCR, respectively. Based on the results, these two SNPs are located just outside the haplotype block showing complete LD with the *Grey* mutation. Similarly, Sanger sequencing of SNP5 and SNP7, located in the vicinity of SNP6, showed that neither one was in complete LD with *Grey*.

In conclusion, this effort to resequence the entire 352 kb region associated with *Grey* and then further genotyping a set of candidate SNPs with strong associations, has provided extensive support for our previous conclusion that the 4.6 kb duplication in *STX17* is the causative mutation for *Grey*. We did not identify any other sequence polymorphism uniquely associated with *Grey* across breeds, where the genotype distribution perfectly matched the Grey phenotype.

### SNP2 is duplicated in the horse genome

Surprisingly, SNP2 did not show any significant linkage disequilibrium to *Grey* at all, in contrast to all other previously analyzed sequence polymorphisms located in the Grey critical interval [2]. Thus, this SNP cannot be involved in determining the Grey phenotype. In TaqMan analysis of this SNP, situated in the 3'UTR of *STX17*, two clearly distinct genotype clusters of individuals that appeared as heterozygous *C/T* were detected, one cluster with a slightly higher *T* signal. This additional cluster contained non-Grey, heterozygous Grey and homozygous Grey individuals, a finding which shows that this polymorphism shows no LD with *Grey* (Table 3). Sanger sequencing confirmed a heterozygous SNP genotype, and also confirmed the higher signal from the *T*-allele in some individuals (data not shown). Furthermore, no individuals homozygous for the *T*-allele were identified in the TaqMan assay, indicating that SNP2 could not be in Hardy-Weinberg equilibrium; the expected number of horses homozygous for the *T*-allele in this material was eight. We hypothesized that this anomalous result could be due to this SNP representing a second transposed copy present somewhere else in the genome. This was confirmed by a PCR-based approach used in order to determine the size of the transposed fragment. We investigated this by amplifying fragments 1 to 5 kb flanking the SNP, followed by SNP genotyping by Sanger sequencing. Since the *T* allele was only detected when the flanking primer was located not more than 1 kb upstream or downstream of the polymorphic nucleotide, we could estimate the size of the transposed fragment to approximately 2 kb and it does not overlap the Grey duplication. Three additional nucleotide positions differing between the *STX17* 3'UTR sequence and the transposed fragment were identified (data not shown). However, we were not able to reach out into unique sequences flanking the transposed fragment by chromosome walking, which would have allowed us to determine the exact genomic position of the second copy of this sequence. An alternative way would be to map the location of this SNP with classical linkage analysis.

In conclusion, our interpretation of this anomalous SNP is that it represents a transposed copy in which the nucleotide corresponding to the C nucleotide at position 6,587,140 bp in *STX17* 3'UTR has been replaced with a T. Animals scored as CC in this assay either lack the transposed copy or carry a transposed copy with a C at this position. The two clusters with different C:T ratios represent horses carrying one or two copies of the transposed sequence with the T nucleotide. The fact that this sequence is located elsewhere in the genome explains the complete lack of linkage disequilibrium with the *Grey* allele (Table 3).

## Discussion

Tandem duplications are notoriously unstable and often show copy number variation [18]. In this study we have explored the possibility that the 4.6 kb duplication causing Greying with age in horses exhibits copy number variation in constitutional and/or tumour DNA. We show that there is a difference in copy number of the Grey duplication between constitutional DNA and tumour DNA, with the highest copy number found in tumours classified as aggressive or derived from horses euthanized possibly due to numerous internal tumours. When screening copy number expansion in constitutional DNA, we found only one horse out of about 90 with an estimated copy number of 5 or higher, whereas the corresponding figure for tumour DNA was 5 out of 8 (P = 5x10^-6^; Fisher’s exact test). The elevated copy number in tumour DNA compared with constitutional DNA constitutes the first set of proof that the duplication in *STX17* not only predisposes to melanoma development, but that copy number expansion of the duplicated sequence may be a driving force in melanoma development. The three horses showing a higher copy number in melanoma tissue than expected from their zygosity for *Grey*, were all euthanized possibly due to what might be internal melanoma metastases (ID1, ID2, ID3; Table 1, Figure 2B). mRNA from the tumour samples ID1 and ID2 were used for expression analysis in our previous study [19] and both samples had upregulated expression of *STX17* and *NR4A3* consistent with our interpretation that the copy number expansion detected in the present study may be relevant for tumour development. The melanoma cell lines showing a higher copy number than expected from the *Grey* genotype, Ho-Mel-A1 (Seltenhammer et al., in preparation) and M14 [20], have both been characterized as malignant, with M14 showing a high expression of the proliferating cell nuclear antigen PCNA [20], previously correlated with aggressive behaviour of human cutaneous malignant melanoma [19].

The present study has provided strong genetic support for our previous conclusion that the 4.6 kb duplication in intron 6 of *STX17* causes Greying with age [19]. Firstly, the copy number expansion seen in tumours suggests that it may promote tumour progression. Secondly, our careful resequencing strategy of the 352 kb region showing complete association with *Grey* failed to identify any other sequence polymorphisms than the duplication that showed a perfect concordance with the phenotype. Furthermore, in a recent study we provided strong support for the functional significance of the duplication [21]. We demonstrate using transfection experiments and transgenic zebrafish that the duplicated sequence contains a melanocyte-specific regulatory element that is MITF (microphtalmia-associated transcription factor)-dependent. The data showed that the duplication transforms a weak enhancer element to a strong melanocyte-specific enhancer [21]. Thus, it is possible that further amplifications of the duplicated sequence, as observed in tumour samples in the present study, promote tumour development.

The great majority of sequence polymorphisms present in domestic animals predate domestication [22]. Thus, most SNP alleles associated with Grey can be expected to be shared with non-Grey haplotypes as demonstrated in this study. However, we identified three nucleotide polymorphisms that were only found in a subset of Grey horses despite the fact that they were located within the region where all sequence polymorphisms show complete LD with *Grey*. Thus, these sequence polymorphisms must have arisen subsequent to the *Grey* mutation. This result is fully consistent with the presumed long history of the *Grey* duplication, with an estimated age older than 2,000 years [2]. This assumption is based on the fact that all tested Grey horses representing breeds from all over the world share the same mutation that must have occurred early during horse domestication to become as widespread as it is. Furthermore, there is abundant written records 2,000 years old and older indicating the occurrence of white horses.

There is a considerable variation in how fast greying proceeds in Grey horses, and in our previous study we demonstrated that Grey homozygotes grey faster than Grey heterozygotes [2]. In this study, we have shown that several late greying Connemara horses have a copy number of three for the *STX17* duplication, as expected for a heterozygous Grey horse (Additional file 2). Additionally, for the investigated SNPs, they proved to have the same haplotype as Grey horses from other breeds. This shows that the late greying phenotype in these horses is caused by the same allele as in other Grey horses, whereas there must be other modifying genetic variation in the vicinity of the *Grey* mutation or elsewhere in the genome.

A surprising finding in this study was that one of the SNPs corresponding to the nucleotide position 6,587,140 bp on horse chromosome 25, i.e. in the region showing complete LD with *Grey*, did not show any signs of LD with *Grey*. We showed that this anomaly occurs because this segment is duplicated in the horse genome and that the polymorphism is derived from the other genomic location explaining the lack of LD with *Grey*. The location of this second copy was not revealed in the present study but it should be straightforward to search for its location using next-generation sequence data in particular since the transposed fragment is only about 2 kb in size. It is possible that the presence of transposed copies of duplicated sequences is an explanation for other similar cases of one or more non-associated SNPs within an ocean of strongly associated SNPs.

## Conclusions

In this paper we show that a 4.6 kb duplication in *Syntaxin 17*, associated with the Grey phenotype in horses, does not only predispose to melanoma development, as previously shown, but that a copy number expansion of the duplication may be a driving force for melanoma development. The extensive genetic analysis, including resequencing of the 352 kb Grey haplotype, confirms that the 4.6 kb duplication is the causative mutation for this phenotype as no other polymorphism showed a complete concordance with the phenotype. The elucidation of the mechanistic features of the duplication will be of considerable interest for the characterization of these horse melanomas as well as for the field of human melanoma research.

## Methods

### Duplication copy number variation analysis by qPCR (TaqMan)

Constitutional DNA was prepared from blood or skin, and tumour DNA was prepared from melanoma cell cultures or melanomas (Table 1) using the DNeasy Blood & Tissue kit (Qiagen, Valencia, CA, USA) according to manufacturer’s protocol. Since the high melanin content in the melanomas can interfere with subsequent applications, the tumour DNA was purified with Chroma Spin TE-1000 columns (Clontech Laboratories Inc., Mountain View, CA, USA) as described in the standard protocol. DNA from the late greying Connemara horses was prepared either from hair by incubating 5–7 hair roots for 60 min at 56°C, in a total volume of 100 μl 5% chelex, containing 140 μg proteinase K, or from 100–200 μl of blood using the E.Z.N.A.® SQ Blood DNA Kit (OMEGA Bio-Tech Inc., Norcross, GA, USA).

Assays for testing the potential copy number variation of the *STX17* duplication and the reference gene assays were designed in PrimerExpress (Applied Biosystems, Foster City, CA, USA). Probes were designed inside and outside the duplicated sequence, and also spanning the 5' flanking sequence and the 5'end of the duplicated sequence and over the duplication breakpoint (Additional file 3). For each region of interest, four replicate CT values were obtained per sample with FAM™ labelled probes for the specific regions and VIC® labelled probes in the reference gene assays. Two reference gene assays, RNaseP and TERT, were tested according to the recommendations by Applied Biosystems, but only the RNaseP assay was used in the analysis since TERT showed signs of amplification or deletion in several samples, as could be expected in cancer cells [23]. TaqMan Copy Number Assays were performed according to the manufacturer's instructions (Applied Biosystems) and using an ABI7900HT instrument. The results were analyzed in CopyCaller™ Software v1.0 (Applied Biosystems). The samples used in the assays consisted of blood DNA from one non-Grey calibrator sample, 32 heterozygous and 62 homozygous Lipizzaner horses, hair DNA from three late greying Connemara horses SP1, SP2 and SP3 (Additional file 2); tumour DNA from four equine melanoma cell lines M1, M14 [20], Ho-Mel-A1 and HO-Mel-L1 (Seltenhammer et al., in preparation), tumour DNA from one heterozygous Grey individual: ID1, and paired constitutional and tumour DNA from one homozygous Grey individual: ID2, and two heterozygous Grey individuals: ID3 and ID4.

### Targeted resequencing

Two individuals were expressly chosen for massively parallel resequencing of the region surrounding the Grey mutation locus. One was a Lipizzaner horse (ID: L147) homozygous for *Grey* (Grey sample). The other was a non-Grey chestnut Arabian horse (ID: 800), with the closely related haplotype on which the Grey mutation occurred (i.e. “ancestral” non-Grey sample). In addition, five other non-Grey horses were included in the experiment: an Appaloosa, an Icelandic, a Knabstrupper, a Noriker and a Quarter Horse. The samples were prepared for sequencing as follows. Genomic DNA was fragmented and purified on QIAquick columns (Qiagen). Fragment ends were blunted and 5' phosphorylated, and a 3' overhang of a single adenosine was added. Illumina adapters (Illumina, San Diego, CA, USA) were ligated to the DNA fragments, which were subsequently hybridized to NimbleGen Sequence Capture Arrays (Roche NimbleGen, Madison, WI, USA), following manufacturer's recommendations. The arrays were designed to target the 352 kb region on horse chromosome 25, harbouring the haplotype previously associated to *Grey*. Following enrichment for target fragments on the arrays, Illumina adapter ligated fragments were amplified with adapter specific primers using a high-fidelity polymerase (Phusion, Finnzymes, Espoo, Finland) and purified on QIAquick columns (Qiagen). The enrichment-factor for each sample was assessed by quantitative PCR comparison to the same samples prior to hybridization. The enriched samples were then sequenced with Illumina Genome Analyzer (Illumina), obtaining single-end 35 bp reads. Mapping of the reads and SNP calling were performed with the standard parameters of the MAQ program, version 0.6.6 [24].

### Genotyping

The inferred genotypes for 10 selected SNPs were confirmed by PCR followed by Sanger sequencing of three individuals; Twilight, the thoroughbred mare that the genome sequence is derived from is heterozygous for *Grey*[17], an homozygous Grey horse (ID: L032) and the individual with a non-Grey haplotype closely related to Grey (ID: 800). Subsequently, TaqMan SNP Genotyping Assays were designed for five SNPs being either polymorphic in the Grey individual or not showing linkage disequilibrium with *Grey* (Figure 3 and Additional file 3). SNPs polymorphic among non-Grey individuals were not considered for further analysis, since we aimed at identifying other mutations associated with the Grey phenotype. In total, 357 Grey and non-Grey horses from 8 different breeds were genotyped for the selected SNPs in order to investigate if any of these SNPs could be associated with the Grey phenotype. Four Connemara horses with the late greying phenotype were included among these 357 horses to investigate possible additional mutations in the Grey haplotype. Samples were genotyped on an ABI7900HT instrument using the TaqMan allelic discrimination assay according to the manufacturer's instructions (Applied Biosystems). Each SNP was genotyped twice for every individual. The SNPs in repetitive regions were amplified by PCR for informative individuals and genotyped by Sanger sequencing (Additional file 3).

The *Grey* mutation was genotyped with long range PCR as previously described [2]. Since this method requires amplification of fragments >4 kb, the DNA isolated from hair samples or old blood samples of late greying Connemara horses could not be used. Therefore, their *Grey* genotypes were deduced from the TaqMan Copy Number Assay.

### Identification of a transposed fragment originating from *STX17* 3'UTR

To identify the approximate size of a duplicated fragment corresponding to the sequence in the *STX17* 3'UTR that could explain the results from the TaqMan SNP screen, PCR products from two individuals genotyped as homozygous *C/C*, two individuals genotyped as *C/T* but with a *C/T*-ratio of 3:1 and two individuals genotyped as *C/T* with a *C/T*-ratio of 2:2 were amplified 5, 4, 3, 2 and 1 kb upstream and downstream of SNP2 (Additional file 3) using the Expand Long Template PCR system Mix 1 (Roche Diagnostics GmbH, Mannheim, Germany), in 25 μl reactions using 125 ng of genomic DNA. The PCR products were treated with *ExoI* and calf intestine alkaline phosphatase (CIAP) in *ExoI* buffer (all from Fermentas, St. Leon-Rot, Germany) at 85°C for 60 min to remove free nucleotides and excess primers prior to sequencing. The SNP was then genotyped by sequencing with primers flanking the polymorphism (Additional file 3). Sequences were analyzed and screened for mutations with Codon Code Aligner (CodonCode Corporation, Dedham, MA, USA).

## Competing interests

A patent has been filed concerning commercial applications of the diagnostic test for the Grey mutation**.** GRP, AG and LA are inventors on this patent application.

## Authors’ contributions

ES participated in the design of the study, carried out the Copy Number Assays, genotyped SNPs and Grey phenotypes, identified the transposed fragment and wrote the manuscript. FI carried out the targeted resequencing and analyzed the results. SM, IC, MHS and JS contributed with material. CW, SS, KLT took part in the targeted resequencing. GRP contributed to the experimental design of the Copy Number Assay. AG took part in the analysis of tumour samples. LA supervised the study, participated in its design and coordination and helped to draft the manuscript. All authors read and approved the final manuscript.

## Supplementary Material

Additional file 1 **Lack of copy number variation outside and over the 5’breakpoint of the STX17 duplication in constitutional DNA from Grey horses.**94 Grey Lipizzaner horses and one calibrator sample with a known copy number of 2 were included in the analysis. OUT = outside the duplicated sequence and BP n = the border between the 5’flanking sequence and the 5’end of the duplicated sequence. Error bars represent the copy number range from the CopyCaller™ Software analysis of quadruplicates in each assay. Click here for file

Additional file 2 **The late greying Connemara horses are heterozygous for the STX17 duplication.**Copy number assay for the STX17 duplication from tests with four different probes using constitutional DNA from the late greying Connemara horses SP1, SP2, SP3. The sample denoted ‘Calibr’ is a g/g individual with a known copy number of 2, used as a calibrator in the IN, OUT and BP n analyses. Constitutional DNA from one G/g and one G/G horse was tested in the assay and the results are shown as a reference for the copy number expected from each genotype. The G/G reference sample was used as a calibrator in the BP d analysis. IN = inside the duplicated sequence, OUT = outside the duplicated sequence, BP n = the border between the 5’flanking sequence and the 5’end of the duplicated sequence and BP d = over the duplication breakpoint. Error bars represent the copy number range from the CopyCaller™ Software analysis of quadruplicates in each assay. Click here for file

Additional file 3 Primer sequences used for characterization of the region harbouring the Grey mutation in horses. Click here for file
