# Supplementary figures and images for: Copy number expansion of the STX17 duplication in melanoma tissue from Grey horses
Source: BMC Genomics. 2012 Aug 2;13:365. doi: 10.1186/1471-2164-13-365 (PMC3443021; doi:10.1186/1471-2164-13-365)

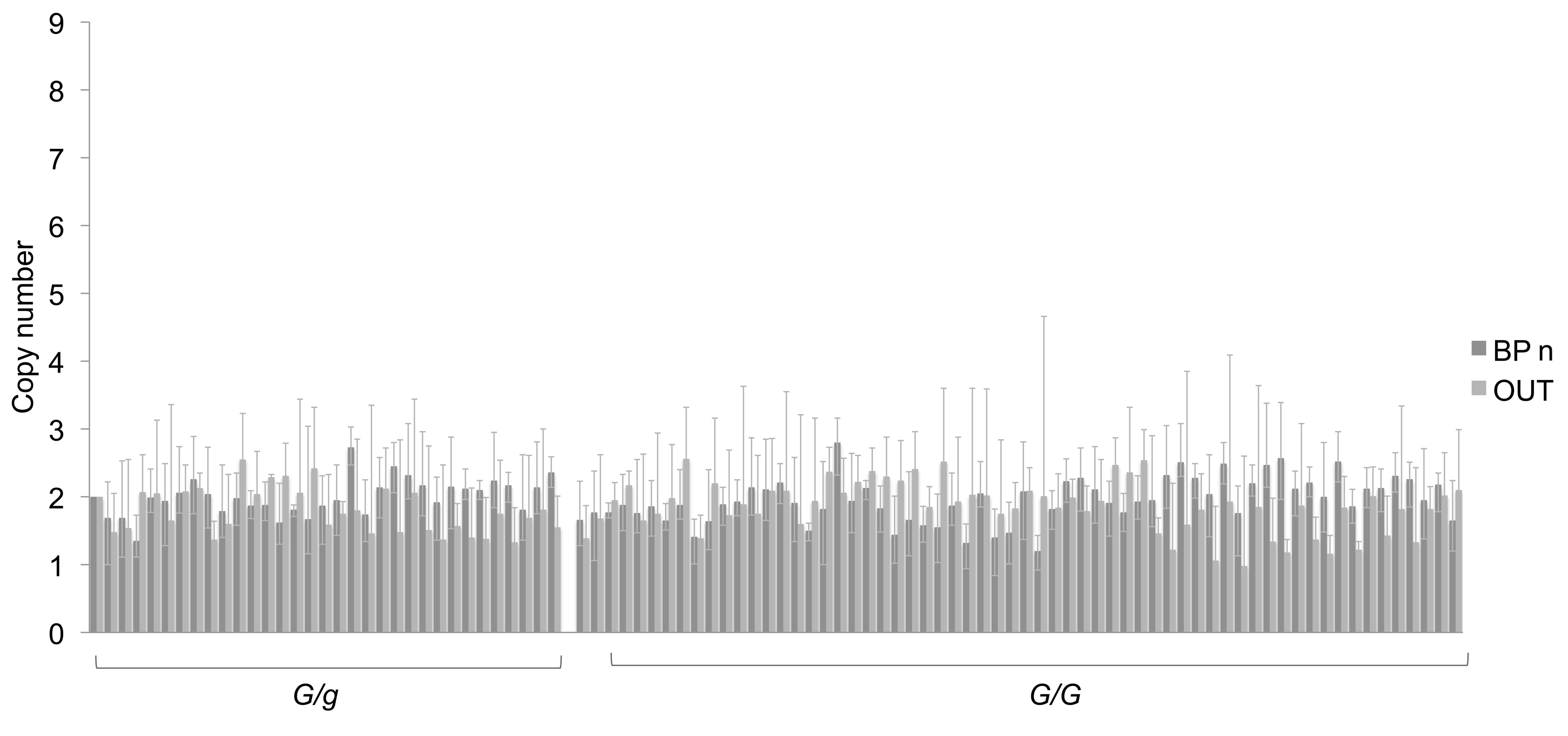

Supplement: Additional file 1 — Lack of copy number variation outside and over the 5’breakpoint of the STX17 duplication in constitutional DNA from Grey horses.94 Grey Lipizzaner horses and one calibrator sample with a known copy number of 2 were included in the analysis. OUT = outside the duplicated sequence and BP n = the border between the 5’flanking sequence and the 5’end of the duplicated sequence. Error bars represent the copy number range from the CopyCaller™ Software analysis of quadruplicates in each assay. [file 1471-2164-13-365-S1.jpeg]

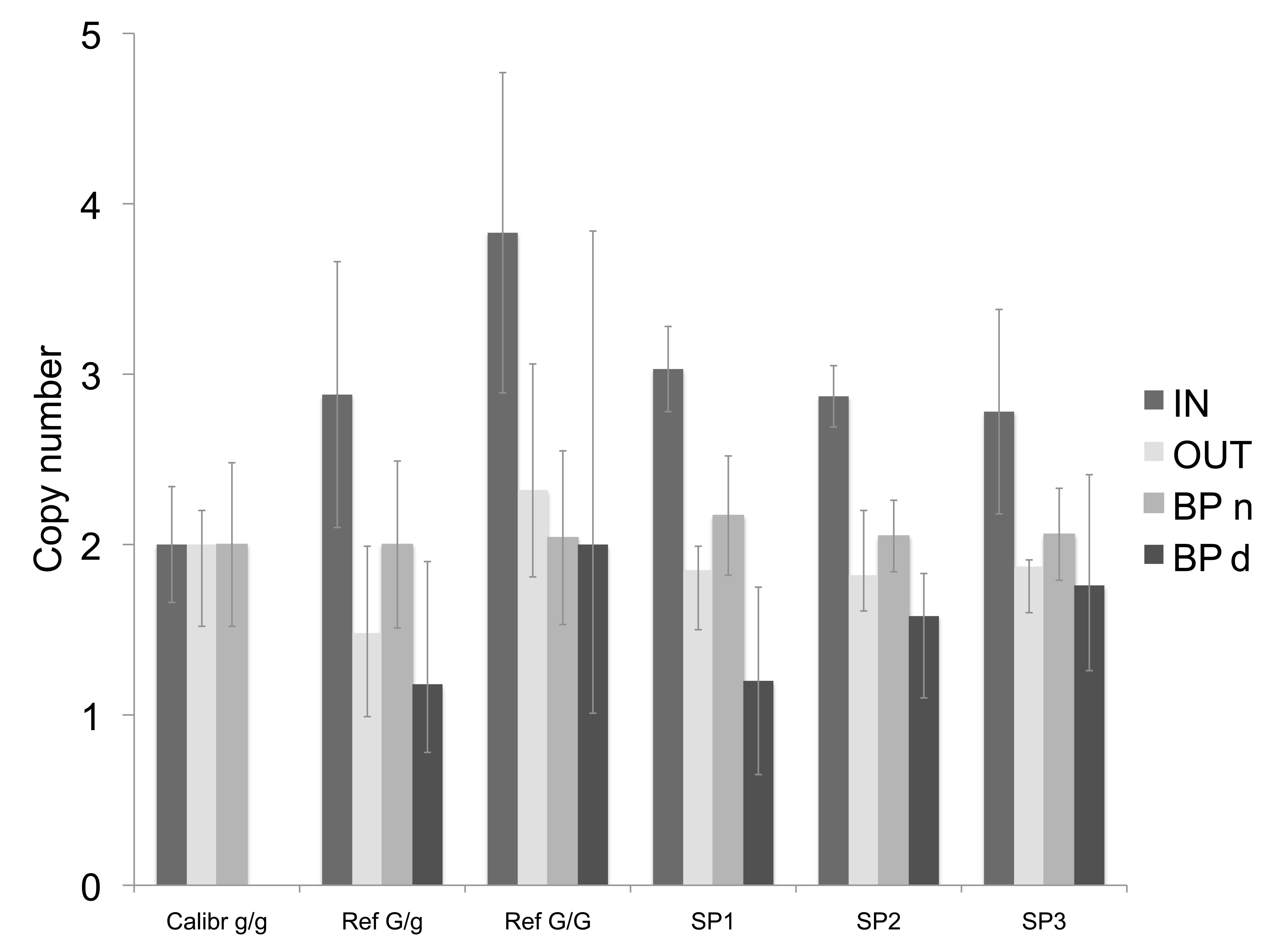

Supplement: Additional file 2 — The late greying Connemara horses are heterozygous for the STX17 duplication.Copy number assay for the STX17 duplication from tests with four different probes using constitutional DNA from the late greying Connemara horses SP1, SP2, SP3. The sample denoted ‘Calibr’ is a g/g individual with a known copy number of 2, used as a calibrator in the IN, OUT and BP n analyses. Constitutional DNA from one G/g and one G/G horse was tested in the assay and the results are shown as a reference for the copy number expected from each genotype. The G/G reference sample was used as a calibrator in the BP d analysis. IN = inside the duplicated sequence, OUT = outside the duplicated sequence, BP n = the border between the 5’flanking sequence and the 5’end of the duplicated sequence and BP d = over the duplication breakpoint. Error bars represent the copy number range from the CopyCaller™ Software analysis of quadruplicates in each assay. [file 1471-2164-13-365-S2.jpeg]
