# Supplementary material for: Copy number expansion of the STX17 duplication in melanoma tissue from Grey horses
Source: BMC Genomics. 2012 Aug 2;13:365. doi: 10.1186/1471-2164-13-365 (PMC3443021; doi:10.1186/1471-2164-13-365)
Supplement: Additional file 3 — Primer sequences used for characterization of the region harbouring the Grey mutation in horses. [file 1471-2164-13-365-S3.docx]

**Additional file 3.** Primer sequences used for characterization of the region harbouring the Grey mutation in horses.

| **Name** | **Forward oligo sequence** | **Reverse oligo sequence** |
| --- | --- | --- |
| **PCR primers** |  |  |
| 6518364 | TGGCATACTCAGAGCTTTCG | TTTTAAGGGTTAAGAGGGAGGAA |
| 6524617 | TTCCTCCTGGCTTCAGTGTT | GTTAACAAAGGGCAGGACCA |
| 6553868 | GGCAAATATATGTACAGCCACTCTT | GGTGACATATCTCTCTCTTTAGTTCTG |
| 6561851 | TCCTCAGGATGGATTTCTGG | CTGGTAATCTCACCCCTCCA |
| 6587140 | TATAGGCTGGCTGTGGCTCT | CCATCCAGCTCTTCCCACTA |
| 6622990 | ACCCCAAAGCTGCAGAATAC | TGGCTGCATGCTGTAAGTTC |
| 6640661 | GACTTGAGAGCAAGGGTAGGG | GACCTTTTCAGGCTCACTGC |
| 6725393 | CAAGGCGTGTTGGAGGTAAT | CCACAGATCACCAGCTCCTT |
| 6753660 | TGTGACGGTGGTTGATGTCT | AAAGTCCTCCTCCCCAAAAA |
| 6778924 | TGGGAGCTGTGCTTACAGTG | TTACTTCCTGCCCATCAAGG |
| 6782059 | GCACTCATCGCCTCTAGACC | CTCTCAGTGGAAGCCACACA |
| 6789297 | AGCTCAGCCAGCAACAATTT | TTGCTGATTTCGCTTGTGTC |
| 6775832 | CATCCGGAATTCATTTTGCT | CCTCACGCAAAGCTATCTCC |
| 6790986 | ACCTGGGCTTACTCATGTGG | GACGCCCTGCTAGGTGTTAG |
| 6792317 | ATCCTGGGCTGCAATGTAAG | ATCTGGATCAGGCTCATTGG |
| 7140_5kbup | TGTGATGAGCGGGTATATTTCGTTGTTT | TGACCACTAAGGTTTACGATTGGGGAAT |
| 7140_4kbup | CCACACAAGGAGTCTCTGACCCTAAGT | TGACCACTAAGGTTTACGATTGGGGAAT |
| 7140_3kbup | TCAGTCCCACGGGAAATTCTAATAATG | TGACCACTAAGGTTTACGATTGGGGAAT |
| 7140_2kbup | GAGGCCAGGATGTTGACTAAGTCTGTT | TGACCACTAAGGTTTACGATTGGGGAAT |
| 7140_1kbup | CTCTGAATCCACAGCCTTCCTCTGT | TGACCACTAAGGTTTACGATTGGGGAAT |
| 7140_5kbdown | GAGGAAGGATGAAGAAGAGCCTGAGAAA | TAGCATCATGGAGCTCAAAGTTGAGTGA |
| 7140_4kbdown | GAGGAAGGATGAAGAAGAGCCTGAGAAA | TTCACCAGGACGAGATGTAAATCAAGA |
| 7140_3kbdown | GAGGAAGGATGAAGAAGAGCCTGAGAAA | CCTTCTTGGGTCCCTACTATTCTGACC |
| 7140_2kbdown | GAGGAAGGATGAAGAAGAGCCTGAGAAA | TCCATCTAACCAGCCTAGTCACTCACA |
| 7140_1kbdown | GAGGAAGGATGAAGAAGAGCCTGAGAAA | AGACGCACTGGTGACAAGACTGATATT |
| Grey_reseq_2059 | CTCACTGTTGCGGGAGTGT | ATGGAGGGAGATGGAAAACC |
| Grey_reseq_8924 | TTTTTCTACTGAGGTAACATTGGTTT | GACCACGGGAGGTAATGGTT |
| Grey_reseq_0986 | AAGGATCGTGTGATGCTTTG | TCTCTGAATCTCAAGCACCTAGC |
| Grey_reseq_7140 | TATAGGCTGGCTGTGGCTCT | CCATCCAGCTCTTCCCACTA |
| Grey_reseq_2059 | TTGTCAGCTCCTTGAGAGCA | CATGGCAATGGACACTTGAA |
| Grey_reseq_3868 | GCCTTTTGATATTGAAATAAGTGTGA | TCACAGTCAGATAAATGGGTCTACA |
|  |  |  |
| **Sequencing primers exclusively** |  |  |
| 0986_F2 | GCCGTTCCTCTACTGGATCA |  |
| 0986_R2 |  | TGTCTCCAGCCCTTGAATCT |
| Grey_reseq_3868_F2 | TGTTTACCAGTGGACTTGTGCT |  |
| Grey_reseq_3868_F3 | AATTAGTCATTTTTCAGAATTTCAGTT |  |
| Grey_reseq_3868_R3 |  | CTGATTTCTTGACAAACACAGAGC |
| Grey_reseq_3868_R2 |  | TGCTGCAATATTCACAGTCAGA |
|  |  |  |
| **Primers in TaqMan SNP assay** |  |  |
| 6553868 | CATAATTAGTCATTTTTCAGAATTTCAGTTAACTCAGAATA | AGAAATATCCTGAAATTCCTGTCTAAAGATTAAAAGA |
| 6587140 | GGATATTATAAACAATCAGTGGTAAACTTAGATCCA | CATAGACGGAGTTCTTGTTTCCCATAA |
| 6640661 | GTGAGAGAAGAGCGATCTGTTCA | TCTGTAATATTCTTTACTCTTTTGTCACTGGAC |
| 6775832 | CCTTTTTCAGATAGGAGCGGGAATT | GTTTCGTTTGCTGGCTCACAA |
| 6778924 | CGTCACCACATGTGCCTAGTC | AAACAAACACATAAATACGCAGATTAGATACG |
| 6782059 | CACTGTAATTTGTCTGCCTCACTCA | AGCATACGTTGTGAGGATAAATGGT |
| 6790986 | GTTAGACCATCTTCCTTTCATGTTGTC | GGAGCTGAGGCCTCAAGAG |
|  |  |  |
| **Probes in TaqMan SNP assay** | **Reporter 1: Dye VIC** |  |
| 6553868_V | AGTACAGATAAGTGT**A**TATTTAA |  |
| 6587140_V | CAGTGATGT**G**GATAATA |  |
| 6640661_V | AAAGAAAATATCAC**G**ACTAAAG |  |
| 6775832_V | CTTTTTTCCCAC**A**TGTCAAA |  |
| 6778924_V | ACCCCTTTT**G**CTCTCC |  |
| 6782059_V | CAGCGAAC**A**CATTTA |  |
| 6790986_V | TGCCTCCTC**A**CTCTCA |  |
|  |  |  |
|  | **Reporter 2: Dye FAM** |  |
| 6553868_M | CAGATAAGTGT**G**TATTTAA |  |
| 6587140_M | CAGTGATGT**A**GATAATA |  |
| 6640661_M | AAGAAAATATCAC**A**ACTAAAG |  |
| 6775832_M | TTTTCCCAC**G**TGTCAAA |  |
| 6778924_M | ACCCCTTTT**A**CTCTCC |  |
| 6782059_M | CAGCGAAC**G**CATTTA |  |
| 6790986_M | TGCCTCCTC**T**CTCTCA |  |
|  |  |  |
| **Primers in TaqMan Copy Number Assay** |  |  |
| Inside duplication | GCATTATTGTTGCTTGATGCAGAGA | GCAGTTTGGGCATCTGAAGTC |
| Outside duplication | GCTTTCGACGTGGTTTGTCA | CCGCAGCCTTGGAGTCTAAC |
| Over normal breakpoint | GAGAAGTTGGGCAAGAGCAGAA | CCTGACATCTGGCTTTAAATTATAAGAA |
| Over duplication breakpoint | GAGAAGTTGGGCAAGAGCAGAA | GGTGGTGCTGATACTACAAGTCCAT |
| RNaseP | GGCGGATGCCTCCTTAGC | TCACCTCAGCCATTGAACTCACT |
|  |  |  |
| **Probes in TaqMan Copy Number Assay** | **Reporter Dye FAM** |  |
| Inside duplication | CTGGGCCTACAAGTGTCCACC |  |
| Outside duplication | ATGCTGTTAATGAACTGC |  |
| Over normal breakpoint | CTGAGATTGTGTGATCTGG |  |
| Over duplication breakpoint | CTGAGATTTTGCATTTCTAATGA |  |
|  |  |  |
|  | **Reporter Dye VIC** |  |
| RNaseP | CTTGGAACAGACTCACGGCCAGCG |  |
